# Lesser Occipital Neuralgia Secondary to Continuous Positive Airway Pressure (CPAP) Mask Headgear: A Case Report

**DOI:** 10.7759/cureus.117419

**Published:** 2026-10-04

**Authors:** Sofia Meixedo, Inácio Menezes, Cláudia Matos Dinis, Pedro M Teixeira, Daniela Martins

**Affiliations:** 1 Department of Physical Medicine and Rehabilitation, Centro de Reabilitação do Norte, Vila Nova de Gaia, PRT; 2 Department of Physical Medicine and Rehabilitation, Unidade Local de Saúde de Viseu Dão Lafões, Viseu, PRT; 3 Department of Rheumatology, Unidade Local de Saúde da Região de Aveiro, Aveiro, PRT

**Keywords:** cervicogenic headache, continuous positive airway pressure (cpap), cpap, external compression headache, nerve block, occipital neuralgia

## Abstract

Occipital neuralgia is a well-characterised cause of unilateral head and neck pain arising from irritation of the greater or lesser occipital nerve (LON). External headwear (helmets, goggles, and tight headbands) is a recognised trigger of pericranial pain, formally classified by the International Classification of Headache Disorders (ICHD-3) as external-compression or external-traction headache. To our knowledge, lesser occipital neuralgia specifically attributed to continuous positive airway pressure (CPAP) mask headgear has not been previously reported. We report an 81-year-old man referred for a one-year history of right-sided cervicalgia and hemicranial headache, initially presumed to be spondylotic given multilevel cervical degenerative disc-osteophyte disease with severe foraminal stenosis on imaging. Only on closer review of the clinical history did the temporal relationship between symptom onset and initiation of nocturnal CPAP for obstructive sleep apnoea, together with hyperalgesia sharply confined to the territory of the LON and pain reproduced by axial rotation, point away from the presumed spondylotic cause and toward mechanical entrapment of the LON by the mask headgear. Pregabalin, physiotherapy, and mesotherapy directed at the presumed spondylotic/myofascial aetiology produced limited benefit. An ultrasound-guided anaesthetic block of the right LON, performed under aseptic technique, resulted in immediate and complete resolution of pain on cervical rotation, confirming the diagnosis. This case supports a mechanical, device-related aetiology of lesser occipital neuralgia in CPAP users, mediated by repetitive strap-related compression/traction over the nerve's course along the posterior border of the sternocleidomastoid muscle, aggravated by cervical rotation, and occurring even in the presence of confounding degenerative cervical spine disease. Clinicians should consider this diagnosis in CPAP users presenting with cervicogenic-type headache, and ultrasound-guided nerve block can serve as both a diagnostic and therapeutic tool.

## Introduction

Occipital neuralgia is defined by the International Headache Society as paroxysmal jabbing pain in the distribution of the greater, lesser, or third occipital nerves, often accompanied by dysesthesia and tenderness over the affected nerve [1]. The lesser occipital nerve (LON) is a cutaneous branch of the cervical plexus (C2, occasionally C3) that hooks around the accessory nerve and ascends along the posterior border of the sternocleidomastoid muscle (SCM) before piercing the deep fascia near the occiput [2]. Because of this close anatomical relationship with the SCM, movements involving contraction of this muscle can dynamically compress or traction the nerve along its course.

Headaches attributed to sustained external pressure on pericranial soft tissue are formally recognised in the International Classification of Headache Disorders (ICHD-3) [1] and have been the subject of increasing clinical interest [3,4], having been described with helmets [5], swimming goggles [6], headbands [7], and more recently N95 respirator straps [8]. Continuous positive airway pressure (CPAP) therapy, the first-line treatment for obstructive sleep apnoea (OSA), is delivered through a mask secured by a multi-point headgear that exerts sustained circumferential tension around the occiput and mastoid region, anatomically overlapping the course of the LON. Despite the widespread use of CPAP, a device-related occipital neuralgia attributable specifically to CPAP headgear has not, to our knowledge, been previously described in the peer-reviewed literature.

We report a case of unilateral cervicalgia and hemicranial headache in a patient with severe, imaging-confirmed cervical spondylotic disease, in whom the spondylotic changes were initially presumed to explain the presentation. It was only through closer re-exploration of the clinical history that a temporal link to nocturnal CPAP initiation emerged, redirecting the diagnostic reasoning toward entrapment of the LON, which was subsequently confirmed and treated by ultrasound-guided nerve block.

## Case presentation

An 81-year-old man was referred to Physical Medicine and Rehabilitation for right-sided cervicalgia and limited cervical rotation. His medical history included cardiovascular risk factors (hypertension, dyslipidaemia, obesity, former smoker), permanent atrial fibrillation anticoagulated with apixaban, OSA treated with a full-face mask nocturnal CPAP, obesity hypoventilation syndrome, chronic obstructive pulmonary disease, gait sequelae of childhood poliomyelitis, and a history of recurrent pericardial effusion secondary to Coxiella burnetii infection treated with a pleuropericardial window. He had no known drug allergies and was largely sedentary. He reported right-sided mechanical-pattern cervical pain, provoked by rightward axial rotation of the neck, with radiation to the ipsilateral temporal region and exacerbation with changes in weather; pain was absent at rest (numerical pain rating scale (NPRS) score of 0/10) and reached 6/10 at its worst, with no pain radiating down the arms, paraesthesia, or other neuropathic features or subjective motor weakness. He had initiated nocturnal CPAP for OSA approximately one year earlier, coinciding with symptom onset. An as-needed combination of a muscle relaxant and the non-steroidal anti-inflammatory drug (NSAID) aceclofenac provided marked, though transient, relief.

Cervical computed tomography (CT) performed in 2022 had shown multilevel degenerative disc-osteophyte disease from C2-C3 to C5-C6 with bilateral neural foraminal narrowing, most marked at C5-C6, and a posterior C5-C6 disc herniation contacting but not compressing the spinal cord; cervical magnetic resonance imaging (MRI) performed in 2025 confirmed marked diffuse facet joint degeneration (notably C1-C2, C3-C4, C4-C5, and C5-C6 on the right, and C2-C3 and C3-C4 on the left), multiple severe foraminal stenoses with probable nerve root compromise (predominantly right-sided), bilateral atlanto-axial joint effusions, and posterior disc bulges at C2-C3, C3-C4, C5-C6, and C6-C7 with mild ventral cord indentation at C3-C4, without abnormal cord signal.

On neurological examination, strength was globally symmetric (Medical Research Council grade 5 [9]), except for grade 4 weakness of shoulder abduction (C5 myotome), with symmetric deep tendon reflexes and no expanded reflexogenic area. Lhermitte's sign was negative, and Spurling's test could not be performed owing to pain-limited cervical rotation. Active cervical range of motion was markedly reduced, with axial rotation of approximately 45° to the left and 20° to the right, extension of 20°, anterior flexion of 15°, and lateral flexion of 20° bilaterally. Pain was reproduced on axial rotation bilaterally, more pronounced to the right. Palpation elicited discomfort over the right levator scapulae insertion and the upper trapezius fibres near the occiput; Tinel's sign over the course of the LON was not elicitable, but there was marked hyperalgesia to light touch over the LON/C3 dermatomal territory, with normal sensation in the upper limbs.

Given the severity of the imaging findings, the working diagnosis at this stage was mechanical neck pain secondary to cervical spondylotic disease, with a possible myofascial component; over the following year, a stepwise conservative approach was pursued accordingly, including a trial of pregabalin 150 mg twice daily, physiotherapy, and mesotherapy targeting a myofascial syndrome of the right levator scapulae and upper trapezius. Each intervention produced only limited symptomatic benefit, and pain reproducible with right axial rotation persisted throughout.

It was only when the persistent, treatment-refractory course prompted a more detailed re-exploration of the clinical history that the true nature of the problem emerged. Symptom onset was found to coincide closely with the initiation of nocturnal CPAP more than a year earlier, and, on renewed examination, the pain and hyperalgesia were sharply confined to the LON/C3 cutaneous territory rather than following a broader radicular distribution. This topographic match between the patient's pain and hyperalgesia and the LON territory, the clear mechanical relationship with cervical rotation (a movement recruiting the SCM), and the temporal association with CPAP mask use - a full-face interface secured by a four-point headgear, with two upper straps arching over the vertex and two lower straps encircling the occipital and upper nuchal region at approximately the level of the mastoid processes - together redirected suspicion toward entrapment of the right LON, as an alternative to the previously presumed C3 radiculopathy secondary to the severe foraminal stenosis on imaging.

After discussion of risks and benefits, and with the patient's informed consent, an ultrasound-guided anaesthetic block of the right LON was performed under aseptic technique. With the patient positioned in the left lateral decubitus position, a high-frequency linear probe was placed transversely over the posterior border of the SCM, using the mastoid process as the proximal bony landmark, at the level of Erb's point, where the LON was identified as a small hyperechoic round structure superficial to the muscle [10]. Using an in-plane, posterior-to-anterior needle approach, 4 mL of an equal-volume mixture of 2% lidocaine and 0.75% ropivacaine was injected around the nerve under direct visualisation, with real-time confirmation of peri-neural spread (Figure 1) without corticosteroid.

**Figure 1:**
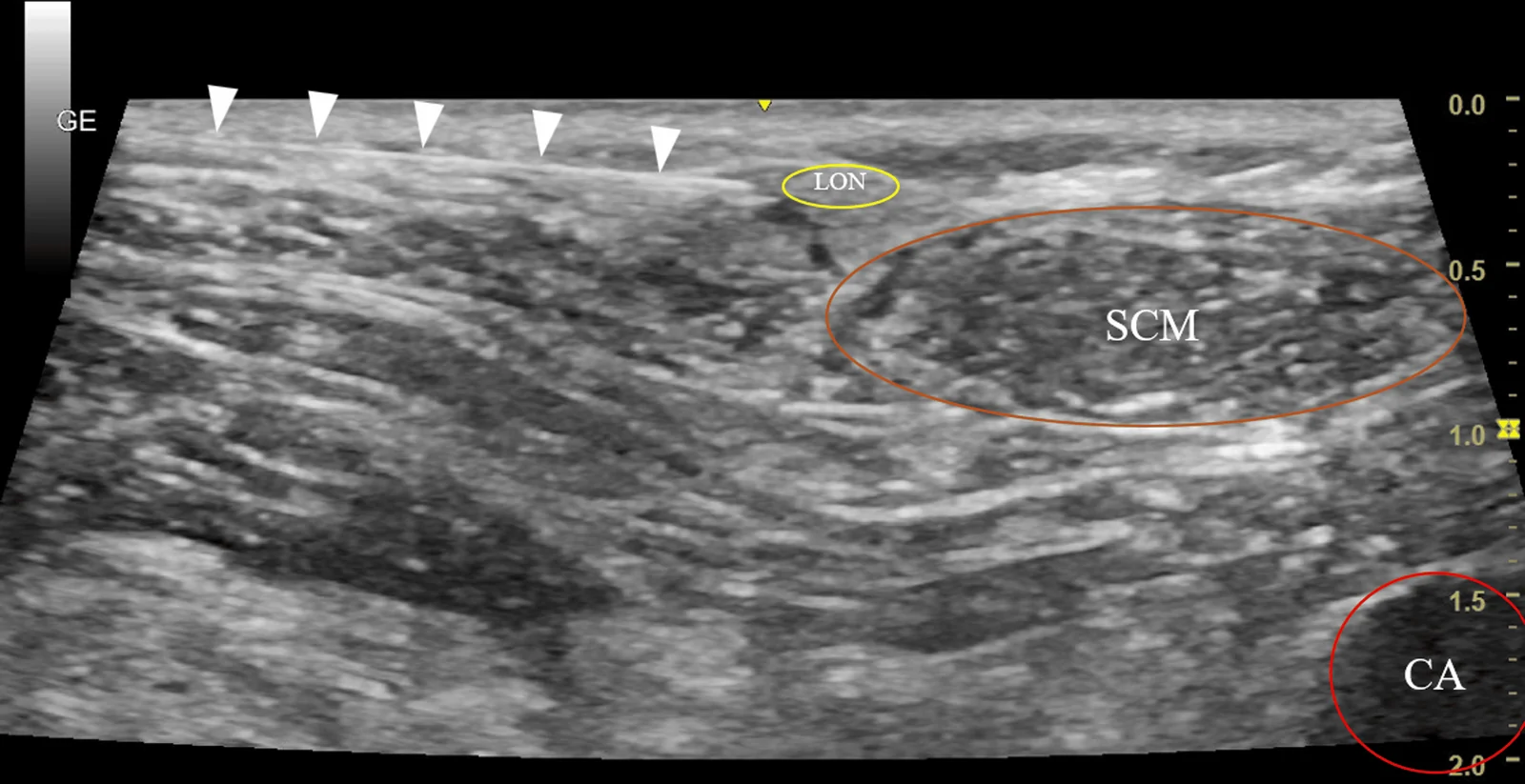
Ultrasound-guided lesser occipital nerve block. White arrowheads indicate the needle trajectory; the LON is outlined by a yellow circle, the SCM by an orange ellipse, and the CA by a red circle. CA, carotid artery; LON, lesser occipital nerve; SCM, sternocleidomastoid muscle

No immediate complications occurred. Immediately after the block, the patient reported complete resolution of the rotation-provoked pain, with NPRS decreasing from 6/10 immediately before the block to 0/10 immediately after, with no residual discomfort on repeat testing. The assisting pulmonologist was advised to evaluate the possibility of changing the patient's CPAP interface.

At the three-month follow-up visit, the patient maintained the benefit obtained with the block and remained asymptomatic, with no recurrence of cervicalgia or hemicranial headache and no further limitation of cervical rotation.

## Discussion

The clinical picture of unilateral cervicalgia and hemicranial headache aggravated specifically by movements recruiting the SCM (axial rotation), with a topography matching the LON distribution, hyperalgesia confined to that territory, and a clear temporal relationship to nocturnal CPAP initiation is most consistent with mechanical irritation of the LON at, or distal to, its emergence at the posterior border of the SCM (Erb's point), where the CPAP headgear strap crosses this territory, and the lower strap is identified as the most anatomically plausible point of contact with the LON's trajectory. This case also illustrates a diagnostic pitfall relevant to everyday practice: the severity of the incidental degenerative imaging findings initially anchored clinical reasoning toward a structural spinal cause, and it was only a deliberate return to the temporal history and to the precise anatomical distribution of the pain that uncovered the coexisting, entirely treatable peripheral entrapment neuropathy.

This mechanism parallels the established construct of external-compression/traction headache, previously described with swimming goggles, helmets, headbands, and respirator masks [3-8], in which sustained pressure or traction from headwear irritates superficial pericranial sensory nerves, including occipital branches. Two features distinguish the presented case from classical external-compression headache: the pain outlasted the period of direct compression (occurring throughout the day, not only during CPAP use), suggesting an evolving focal neuropathy rather than a purely mechanical, fully reversible phenomenon, and the diagnosis was confirmed pharmacologically, via immediate and complete resolution of the rotation-provoked pain after ultrasound-guided anaesthetic block of the LON - a technique with established diagnostic and therapeutic value in occipital neuralgia and cervicogenic headache [11,12].

A relevant confounder in this case was the presence of severe, multilevel cervical spondylotic disease with foraminal stenosis, which raised C3 radiculopathy as a plausible alternative or additional contributor to the patient's symptoms. However, several clinical features support a peripheral LON-mediated component to the patient's symptoms: the hyperalgesia was sharply confined to the LON/C3 cutaneous territory rather than following a broader radicular pattern; upper-limb strength and sensation were otherwise preserved (aside from an isolated, likely incidental, C5 myotome weakness), and the complete resolution of the rotation-provoked pain followed a peripheral nerve block rather than requiring a root-level or central intervention. This does not exclude a contribution from cervical spondylosis to the patient's broader mechanical neck pain, but it does support the LON entrapment as the principal generator of the rotation-triggered, hemicranial component of his presentation.

The dynamic component of the presentation is, to our knowledge, a novel observation in the context of device-related occipital neuralgia and highlights the biomechanical interaction between an external strap and an underlying muscle whose contraction displaces the nerve against that same strap. It may also explain why conservative measures directed at myofascial pain (mesotherapy, physiotherapy) and centrally acting neuropathic agents (pregabalin) achieved only partial benefit: none of these interventions specifically addressed the mechanical strap-nerve interaction.

Clinically, this case underscores three points: occipital neuralgia should be considered in the differential diagnosis of new-onset cervicalgia or hemicranial headache in CPAP users, particularly when pain is reproducible with cervical rotation; ultrasound-guided LON block is a low-risk, high-yield diagnostic and therapeutic option, even in the presence of confounding degenerative spine disease; and headgear refitting or repositioning may be a simple preventive measure once the diagnosis is established.

As limitations, the authors must acknowledge that, as a single case report, causality cannot be established with certainty, and natural history without intervention remains undetermined. The coexistence of severe cervical spondylosis limits definitive attribution of all the patient's neck symptoms to the LON alone. Longer follow-up and larger case series or prospective observation in CPAP clinics would help establish the true incidence of this presentation.

## Conclusions

Based on this case, CPAP headgear may represent a potential mechanical contributor to lesser occipital neuralgia, particularly in patients reporting cervicalgia or hemicranial headache aggravated by cervical rotation, even when confounding degenerative cervical spine disease is present. Ultrasound-guided nerve block offers both diagnostic confirmation and symptomatic relief, and awareness of this entity may prompt earlier recognition and simple preventive strategies such as headgear adjustment.
